# The efficiency of indicator groups for the conservation of amphibians in the Brazilian Atlantic Forest

**DOI:** 10.1002/ece3.1073

**Published:** 2014-05-21

**Authors:** Felipe Siqueira Campos, Joaquim Trindade-Filho, Daniel Brito, Gustavo A Llorente, Mirco Solé

**Affiliations:** 1 Departament de Biologia Animal (Vertebrats), Facultat de Biologia, Universitat de BarcelonaBarcelona, ES-08028, Spain; 2 CAPES Foundation, Ministry of Education of BrazilBrasília, DF, 70040-020, Brazil; 3 Programa de Pós-Graduação em Ecologia e Conservação da Biodiversidade, Universidade Estadual de Santa CruzIlhéus, BA, 45662-000, Brazil; 4 Programa de Pós-Graduação em Ecologia e Evolução, Universidade Federal de GoiásGoiânia, GO, 74001-970, Brazil

**Keywords:** Amphibians, Atlantic Forest, biodiversity indicators, representativeness, surrogates, systematic conservation planning

## Abstract

The adequate selection of indicator groups of biodiversity is an important aspect of the systematic conservation planning. However, these assessments differ in the spatial scales, in the methods used and in the groups considered to accomplish this task, which generally produces contradictory results. The quantification of the spatial congruence between species richness and complementarity among different taxonomic groups is a fundamental step to identify potential indicator groups. Using a constructive approach, the main purposes of this study were to evaluate the performance and efficiency of eight potential indicator groups representing amphibian diversity in the Brazilian Atlantic Forest. Data on the geographic range of amphibian species that occur in the Brazilian Atlantic Forest were overlapped to the full geographic extent of the biome, which was divided into a regular equal-area grid. Optimization routines based on the concept of complementarily were applied to verify the performance of each indicator group selected in relation to the representativeness of the amphibians in the Brazilian Atlantic Forest as a whole, which were solved by the algorithm “simulated annealing,” through the use of the software MARXAN. Some indicator groups were substantially more effective than others in regard to the representation of the taxonomic groups assessed, which was confirmed by the high significance of the data (*F* = 312.76; *P* < 0.01). Leiuperidae was considered as the best indicator group among the families analyzed, as it showed a good performance, representing 71% of amphibian species in the Brazilian Atlantic Forest (i.e., 290 species), which may be associated with the diffuse geographic distribution of their species. In this sense, this study promotes understanding of how the diversity standards of amphibians can be informative for systematic conservation planning on a regional scale.

## Introduction

Increased rates of habitat loss and human occupation are creating demands for more adequate strategies to maximize efforts for biodiversity conservation (Diniz-Filho et al. 2008). One of the conservation strategies mostly used to preserve threatened species is the establishment of protected areas (Lawler and White 2008). The selection of sites for the protection of biological communities and the maintenance of ecosystem processes, within the context of systematic conservation planning (see Margules and Pressey 2000), is an extremely efficient tool to preserve species and habitats (Clemens et al. 1999; Myers et al. 2000; Kati et al. 2004; Rodrigues and Brooks 2007; Loucks et al. 2008). However, the resources available for the creation of protected areas are limited (Loucks et al. 2008). Therefore, it is no surprise that the inclusion of the economic costs into conservation planning can result in more feasible conservation strategies on the ground (Naidoo et al. 2006).

A central issue in systematic conservation planning is the identification of targets to be conserved (Margules and Pressey 2000; Groves et al. 2002; Cowling and Pressey 2003; Sarkar 2004). Protected area networks are often selected to protect species of distinct taxonomic groups, communities of high biological relevance, or combinations of different abiotic conditions favorable to local ecosystems, with the assumption that such sites will also protect a wider range of biodiversity (Lawler and White 2008). Therefore, conservation planners should count on surrogates, or indicator groups, to represent the largest possible part of local biodiversity in reserve selection (Kremen 1992; Raven and Wilson 1992; Flather et al. 1997). The validity of this hypothesis depends on how well the chosen indicator group represents a wider array of biodiversity (Lawler and White 2008). In this way, the adequate selection of indicator groups is fundamental for the consistency of successful systematic conservation planning (Margules and Pressey 2000; Margules and Sarkar 2007).

Most conservation plans are based on the biodiversity surrogates (e.g., Loiselle et al. 2003; Stoms et al. 2005; Margules and Sarkar 2007; Rodrigues and Brooks 2007). These surrogates are generally based on the species, such as keystone species, umbrella species, or flagship species (Andelman and Fagan 2000; Mace et al. 2007; Grantham et al. 2010). Additionally, these surrogates may also be based on other parameters, such as vegetation structure, soil coverage, and environmental gradients (Faith and Walker 1996a,b1996b; Sarkar et al. 2005; Trakhtenbrot and Kadmon 2005), even though it is known that surrogates based on the species are more efficient than those based on environmental proxies (Rodrigues and Brooks 2007).

Quantifying the spatial congruence between species richness and complementarity among different taxonomic groups is a fundamental step to identify potential indicator groups (Howard et al. 1998; van Jaarsveld et al. 1998; Pinto et al. 2008). However, these evaluations differ in spatial scale, in the methods used and in the groups that are tested, which generally produces contradictory results (e.g., Schmit et al. 2005; Bani et al. 2006; Lamoreux et al. 2006; Chiarucci et al. 2007; Rodrigues and Brooks 2007; Grantham et al. 2010; Lewandowski et al. 2010). In spite of the importance and usefulness of systematic investigations about the consistency of indicator groups to guide conservation actions and decision-making processes, only a few studies have explicitly evaluated this aspect (e.g., Araújo et al. 2001; Manne and Williams 2003; Bani et al. 2006; Lawler and White 2008; Trindade-Filho and Loyola 2011).

There is a trend in the scientific literature in relation to studies on organisms that indicate habitat quality (Lima 2001). In this sense, amphibians have been identified as potential biological indicators due to their naked skin and their use of aquatic and terrestrial habitats, which makes them extremely vulnerable to environmental disturbances (Blaustein and Wake 1995; Tocher et al. 1997; Cosson et al. 1999; Kwet and Di-Bernardo 2002; DeGarady and Halbrook 2006; Lebboroni et al. 2006). However, these previous studies did not clearly evaluate which characteristics might make amphibians a good indicator group across different taxa (Sewell and Griffiths 2009). This suggests that some taxa previously highlighted as good indicators could have appeared so simply because they harbored many species, instead of really exhibiting good indicator qualities (Larsen et al. 2009). In order to use a straightforward approach to improve this concept, the main purpose of this study was to assess the performance of amphibian families as potential indicator groups to represent overall amphibian diversity in the Brazilian Atlantic Forest.

## Materials and Methods

### Study area

The Brazilian Atlantic Forest was chosen as our case study because it is one of the 34 global biodiversity hotspots for conservation priorities (Mittermeier et al. 2004), having high rate of habitat loss (Teixeira et al. 2009), which is one of the main factors that driving amphibians to extinction (Stuart et al. 2004; Becker et al. 2007). This biome originally covered approximately 150 million hectares, but it is now reduced to only 11.4–16.0% of its pristine cover (Ribeiro et al. 2009). The majority of the forest remnants cover less than 100 hectares (Ranta et al. 1998) and are isolated from each other, representing forests at early and middle succession stages (Viana et al. 1997; Metzger 2000; Metzger et al. 2009). The remaining large fragments are located in hilly terrain, hindering human occupation (Silva et al. 2007). Yet, the ranges of different altitudinal and latitudinal gradients where these remnants are found have favored a high biodiversity as compared to other biomes in Brazil (Ribeiro et al. 2009).

The Atlantic Forest is the leader biome in amphibian diversity in Brazil, comprising about 400 species (i.e., about 50% of all amphibian species within Brazil, Haddad et al. 2008). This high species richness is explained by the high diversity of habitats and microhabitats, which favor endemisms (Haddad 1998).

### Data

Data on the geographic range of Atlantic Forest amphibian species were obtained from the IUCN Red List of Threatened Species database (IUCN 2012). The software ArcGIS 9.3 (ESRI 2008) was used to overlap the species ranges to the full geographic extent of the biome, which was divided into a regular equal-area grid containing cells with spatial resolution of 0.5° (i.e., about 50 km^2^), providing a network of 436 cells. The total land area covered by this grid was based on the atlas of the remaining Atlantic Forest (SOS Mata Atlântica and Instituto Nacional de Pesquisas Espaciais 2008).

Presence–absence data matrices were designed for 408 amphibian species occurring in the Brazilian Atlantic Forest in such a way that a given species was considered as present when its area of occurrence included any section of the grid system.

Species were divided into eight potential indicator groups, which were based on the different taxonomic groups represented by the families Brachycephalidae, Bufonidae, Cycloramphidae, Hylidae, Hylodidae, Leiuperidae, Leptodactylidae, and Microhylidae. Amphibian families with less than 20 species were excluded from the analyses because of their small sample size. These families included the Allophrynidae, Aromobatidae, Caeciliidae, Centrolenidae, Ceratophryidae, Craugastoridae, Dendrobatidae, Eleutherodactylidae, Hemiphractidae, Pipidae, Ranidae, Plethodontidae, Rhinatrematidae, and Strabomantidae. The taxonomy adopted for the families followed the classification proposed by Blackburn and Wake (2011).

### Analyses

In order to evaluate the performance of indicator groups (amphibian families), the smallest set of grid cells needed to represent all species of each indicator group was selected to solve a problem known as “minimum set coverage” (Underhill 1994). Then, the species representation was maximized with the lowest possible number of cells (Church et al. 1996; Andelman et al. 1999; Cabeza and Moilanen 2001). Thus, a set of eight cells was chosen as the lowest number of cells needed to represent all species among the potential indicator groups assessed.

After that, the 20 best sets of solutions to maximize the representation of each indicator group within eight cells were selected, solving the problem known as “maximal representation problem” (Church et al. 1996). The best spatial solutions to represent the maximum number of species in each group were encountered, with the condition that these solutions do not exceed a set of eight cells in the grid system. This was necessary to evaluate the effectiveness of the selected indicator groups (i.e., the percentage of diversity represented), so they could be compared without biases related to the number of cells contained in each group (see Lawler and White 2008).

Optimization routines based on the concept of complementarity (Vane-Wright et al. 1991; Howard et al. 1998; Cabeza and Moilanen 2001) were then used to verify the performance of each indicator group in regard to the representativeness of overall amphibian species. This concept assumes a nonoverlapping representation of natural features (Cabeza and Moilanen 2001), providing a measure of the contribution of an area to the full complement of biodiversity features assessed (Margules and Sarkar 2007), which implies that the conservation benefits that follow from a particular conservation action at a site depend on the regional context of the site and conservation actions taken elsewhere (Moilanen 2008). Optimization problems were solved by the algorithm “simulated annealing” (Kirkpatrick et al. 1983; Possingham et al. 2000), which was run 10,000 times for each group, using the software MARXAN, version 2.43 (Ball et al. 2009). This is a nonsequential algorithm that looks for optimal solutions (minimum number of cells) by comparing entire sets of areas. Initially, the algorithm selects a random network of cells and, at each iteration (in this case, 10,000 iterations), it randomly changes the system by adding, deleting, and/or switching cells (Possingham et al. 2000) and thus compares the changes resulting in a cost equation (Kelley et al. 2002). The increased acceptable cost decreases at each iteration (Andelman et al. 1999). Therefore, at each step, the new solution is compared with the former solution and the best one is maintained (Kirkpatrick et al. 1983; Possingham et al. 2000).

The average conservation percentage of target species represented a measure of the performance of each indicator group selected. For comparison, 20 solutions were tested with the smallest set of grid cells required to represent all species of each indicator group based on a random collection of species, assessing their effectiveness in relation to all studied species. These sets were built to evaluate whether the performance of the selected indicator groups was higher, similar, or lower than that expected randomly, extrapolating the representation of a null model.

In addition, land cost-effective relationships were calculated according to the number of grid cells required to represent all species from each indicator group assessed. The land cost-effective values were based on the model proposed by Bode et al. (2008), which established an economic cost of 68,733 dollars by each km^2^ of Brazilian Atlantic Forest. Thus, it was possible to provide an economic cost estimation of the minimum effective land coverage of each indicator group.

The relationship between the number of species and the representativeness of each indicator group evaluated was correlated by linear regression analyses, using the software Ecosim 7.72 (Gotelli and Entsminger 2005). Subsequently, the average representation percentage of each indicator group was compared through an analysis of variance (ANOVA), using the software STATISTICA, version 8.0 (StatSoft, Inc 2007), where the effectiveness in capturing biodiversity represented by the relative number of species recorded was the response variable. The significance level of this analysis was 1% because even though the sets of solutions for each indicator group are unique, there may be a large overlay of the cells regarded as important, therefore reducing the independence of solutions (Lawler and White 2008). Diminishing the significance level to a more conservative value may be a way to reduce the effects of spatial autocorrelation when specific methods to control this phenomenon are not applicable or are simply unnecessary (Diniz-Filho et al. 2003; Kubota et al. 2007; Loyola 2009; Trindade-Filho and Loyola 2011).

## Results

### Spatial patterns of species richness

**Figure 1:**
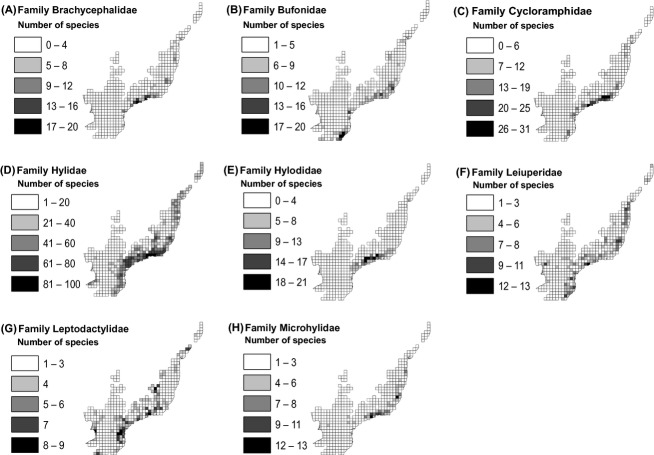
Spatial patterns of species richness from eight potential indicator groups assessed in the Brazilian Atlantic Forest (*n* = 408 species). (A) Number of Brachycephalidae species. (B) Number of Bufonidae species. (C) Number of Cycloramphidae species. (D) Number of Hylidae species. (E) Number of Hylodidae species. (F) Number of Leiuperidae species. (G) Number of Leptodactylidae species. (H) Number of Microhylidae species.

The geographical distribution of the eight potential indicator groups showed different spatial patterns of species richness among them (Fig. 1). There was greater species richness in the southeastern Brazil, mainly for Brachycephalidae, Cycloramphidae, Hylidae, Hylodidae, and Microhylidae. However, Hylidae, Leiuperidae, and Leptodactylidae also were well represented within the southern and northeastern regions (Fig. 1), so that Bufonidae was more distributed in the southern and southeastern Brazil (Fig. 1).

### Performance and efficiency of indicator groups

**Figure 2:**
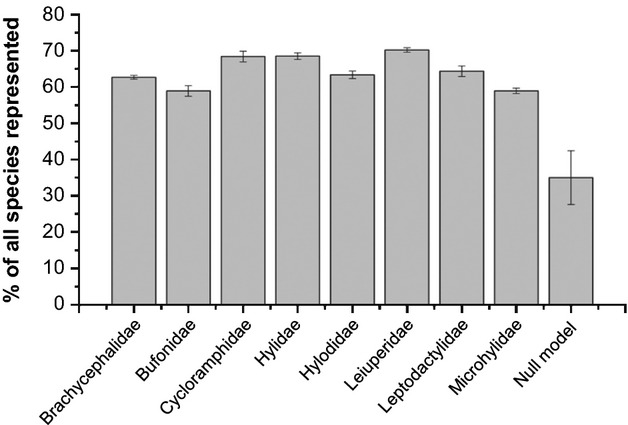
Efficiency of indicator groups to represent the amphibian species in the Brazilian Atlantic Forest. Gray bars represent the mean percentages among the 20 best solutions to represent all species as from the smallest set of grid cells necessary for each indicator group. Error bars denote standard deviations of the means.

The use of families as overall amphibian diversity indicators represented more species than the random choice for representative areas of amphibian diversity in the Brazilian Atlantic Forest (Fig. 2). All amphibian family groups analyzed were considered as potential indicators and showed a good spatial congruence in relation to their representativeness, because all the groups considered individually accounted for more than 50% of the species pool assessed (Fig. 2, Table 1). However, some indicator group indicators were more effective than others in regard to the representation of the taxonomic groups assessed (*F* = 312.76; *P* < 0.01). Leiuperidae was considered as the best indicator group, as it showed a good performance and cost-effective, representing 71% of amphibian species in the Brazilian Atlantic Forest (i.e., 290 species) from only eight grid cells, being based on a group with a relatively low number of species (i.e., 31 species; Fig. 2, Table 1). Species richness within the indicator groups was not correlated with the mean representativeness among them (*r* = 0.40; *P* > 0.15; see Table 1).

**Table 1:** Number of species, number of grid cells required to represent all species, percentage of species represented, and land cost-effective by each indicator group assessed in the Brazilian Atlantic Forest

Indicators Groups (IG)	Number of species per IG	Number of grid cells required to represent all species from each IG	Percentage of species represented by IG (%)	Land cost-effective by IG ($)
Brachycephalidae	35	9	63	30,929,850
Bufonidae	33	9	59	30,929,850
Cycloramphidae	41	11	69	37,803,150
Hylidae	184	26	69	89,352,900
Hylodidae	33	13	65	44,676,450
Leiuperidae	31	8	71	27,493,200
Leptodactylidae	30	11	65	37,803,150
Microhylidae	21	8	59	27,493,200

## Discussion

One of the biggest challenges for tropical conservation biology is to develop precise methods for conservation planning (Becker et al. 2010). Our results indicate that sites selected from potential indicator groups can include a large part of the diversity of amphibians in the Brazilian Atlantic Forest. Similar conclusions were obtained using similar methodologies applied to other taxonomic groups (e.g., Lawler et al. 2003; Loyola et al. 2007; Lawler and White 2008; Pinto et al. 2008; Larsen et al. 2009; Trindade-Filho and Loyola 2011; Trindade-Filho et al. 2012), even though their results can be considered controversial (see Lawler et al. 2003). Some authors have argued that the efficient use of indicator groups requires the selection of large extensions of land, so that the majority of the target species can be represented (see Howard et al. 1998). However, our results showed that good indicator groups can effectively represent biodiversity from a relatively small area.

A species taxonomic group can be considered a good indicator when its geographic distribution spatially coincides with the distribution of the other groups in a given region (Gaston 1996; Flather et al. 1997; Virolainen et al. 2000). In regard to amphibians, although they have been widely promoted as indicators of environmental quality, rigorous complementarity tests are still lacking (Sewell and Griffiths 2009). In large spatial scales, the objective is not to identify areas for protected areas, but to identify regions of high value for conservation that are important in the scale in question (Moore et al. 2003). Besides representing all conservation targets, the regions selected by complementarity are constituted by the lowest possible pool of cells (i.e., minimum of resources) (Lawler et al. 2003).

The performance observed for Leiuperidae as an indicator group may be associated with the diffuse geographic distribution of their species, the lower number of grid cells required to represent all of the species of each indicator group, and the low number of species which compose this group in comparison with the other groups evaluated (see Table 1). Leiuperidae species cover a wide range of different environmental conditions (Grant et al. 2006), representing a great spatial heterogeneity. These species co-occur in common habitats as much for generalist species as for specialist species, providing the occurrence of complementary groups, which favors a greater beta diversity (Loyola et al. 2007; Lawler and White 2008; Pinto et al. 2008; Larsen et al. 2009; Trindade-Filho and Loyola 2011). However, some authors argue that only species with restricted distribution exhibit congruent geographic standards compared with other species distributed in wide spatial scales (Lamoreux et al. 2006).

Our results are relatively optimistic, because they consist of a representation of species in at least one grid cell. This is a limitation, because restricting species occurrence to a single site is similar of the old adage of putting all your eggs on a single basket (see Ricketts et al. 2005). Conservation outcomes were most sensitive to uncertainty in the land cost data, because the use of species extents of occurrence overestimates their real geographic ranges (Rondinini et al. 2006), which in turn increase the effectiveness of indicator groups whose distribution was based on such maps. One possible solution would be the utilization of species distribution modeling methods currently available (Araújo and New 2007). However, these models are known have other sources of uncertainties (Loiselle et al. 2003; Wilson et al. 2005; Diniz-Filho et al. 2009a,b2009b, 2010). Nevertheless, as we are not proposing the creation of protected areas, but suggesting that the use of indicator groups to operate as a shortcut for mapping biodiversity, the use of species extents of occurrence may still be considered a possible solution to investigate the efficacy of indicator groups (e.g., Lawler et al. 2003; Loyola et al. 2007; Rodrigues and Brooks 2007; Lawler and White 2008; Pinto et al. 2008; Larsen et al. 2009; Grantham et al. 2010; Trindade-Filho and Loyola 2011; Trindade-Filho et al. 2012).

For this purpose, future studies on species inventories could be concentrated on the groups scientifically proven as indicators of biodiversity. This suggests that taxonomists tend to concentrate their efforts in the localities that guarantee success in the collection of as many species as possible (Sastre and Lobo 2009). Optimal solutions of complementarity based on different biodiversity analyses have been successful in conservation planning at the global level (Csuti et al. 1997), including for amphibians (Diniz-Filho et al. 2006). The use of taxonomic subgroups as potential indicators of biodiversity has also been a common practice in conservation studies (e.g., Simberloff 1998; Caro and O'Doherty 1999; Andelman and Fagan 2000). In this context, biodiversity surrogate groups and indicator groups have been utilized in different ways to guide conservation strategies (Caro and O'Doherty 1999). Yet, there is an ample spectrum of circumstances that define the relative complexity of conservation planning based on the use of indicator groups (Stoms et al. 2005). Indicator groups should follow predictors of complementarity performance, such as variability between extents of occurrence, occupation of different ecoregions, variability of records of geographic distribution, and average body size in relation to the species pool considered in the analyses (Manne and Williams 2003).

Nevertheless, when we try to choose a specific target to protect other biodiversity aspects than species richness, we create a challenge to the conservation biologists. Here, we are proposing that the use of amphibian families as indicator groups of biodiversity can be a straightforward strategy to maximize the conservation value of small spatial scales. Usually, we must allocate conservation efforts to areas with higher diversity than expected by chance. However, this depends on the purpose of the conservation plan as well on the nature of the ecosystem we are interested in protect. In practice, our results carry a great deal of interest, not only because they are novel, but also because they reveal that a taxonomically defined group (i.e., Leiuperidae) can be used as a conservation shortcut of amphibian biodiversity in the Brazilian Atlantic Forest.

Even though the indicator groups presented in this study had a good performance in representing amphibian diversity in the Brazilian Atlantic Forest, it is important to note that our analyses evaluated efficacy based on a single measurement of diversity. Therefore, we did not incorporate other important aspects, such as population viability (see Carroll et al. 2003), functional diversity, and phylogenetic relationships (see Carvalho et al. 2010; Devictor et al. 2010; Trindade-Filho et al. 2012). However, this was due to the limited knowledge about the majority of the species of our data group. A recent analysis showed that the data-deficient species also seems to reflect a spatial knowledge deficiency (Brito 2010). This lack of knowledge underscores the urgent need for the development of strategies toward systematic conservation planning, which may contribute directly to the stability of the ecosystems and long-term evolutionary processes (Trindade-Filho et al. 2012). In this sense, this study helps in understanding how the spatial patterns of amphibians can be informative for the conservation planning at regional scales.
